# A Recombinant System and Reporter Viruses for Papiine Alphaherpesvirus 2

**DOI:** 10.3390/v14010091

**Published:** 2022-01-05

**Authors:** Abdul Rahman Siregar, Sabine Gärtner, Jasper Götting, Philipp Stegen, Artur Kaul, Thomas F. Schulz, Stefan Pöhlmann, Michael Winkler

**Affiliations:** 1 German Primate Center, Infection Biology Unit, Leibniz Institute for Primate Research, 37077 Gottingen, Germany; ASiregar@dpz.eu (A.R.S.); sgaertner@dpz.eu (S.G.); philipp.stegen@outlook.com (P.S.); akaul@dpz.eu (A.K.); spoehlmann@dpz.eu (S.P.); 2 Faculty of Biology and Psychology, University Göttingen, 30073 Gottingen, Germany; 3 Faculty of Biology, Universitas Gadjah Mada, Yogyakarta 55281, Indonesia; 4 Institute of Virology, Hannover Medical School, 30625 Hannover, Germany; Goetting.Jasper@mh-hannover.de (J.G.); Schulz.Thomas@mh-hannover.de (T.F.S.)

**Keywords:** herpesvirus, Papiine alphaherpesvirus 2, fosmid, transformation-associated recombination, recombineering, reporter virus, Gaussia luciferase, cell susceptibility, antiviral, neutralization

## Abstract

Primate simplex viruses, including Herpes simplex viruses 1 and 2, form a group of closely related herpesviruses, which establish latent infections in neurons of their respective host species. While neuropathogenic infections in their natural hosts are rare, zoonotic transmission of Macacine alphaherpesvirus 1 (McHV1) from macaques to humans is associated with severe disease. Human infections with baboon-derived Papiine alphaherpesvirus 2 (PaHV2) have not been reported, although PaHV2 and McHV1 share several biological properties, including neuropathogenicity in mice. The reasons for potential differences in PaHV2 and McHV1 pathogenicity are presently not understood, and answering these questions will require mutagenic analysis. Here, we report the development of a recombinant system, which allows rescue of recombinant PaHV2. In addition, we used recombineering to generate viruses carrying reporter genes (Gaussia luciferase or enhanced green fluorescent protein), which replicate with similar efficiency as wild-type PaHV2. We demonstrate that these viruses can be used to analyze susceptibility of cells to infection and inhibition of infection by neutralizing antibodies and antiviral compounds. In summary, we created a recombinant system for PaHV2, which in the future will be invaluable for molecular analyses of neuropathogenicity of PaHV2.

## 1. Introduction

Simplex viruses are a subgroup of herpesviruses, which have coevolved with their respective host species [1,2]. After acute infection, they establish lifelong latency in neuronal ganglia of their natural host, from which reactivation and shedding can occur [1,3]. Neuropathogenicity in their respective host species is rare. However, upon cross-species transmission, severe infections of the CNS leading to encephalitis and ultimately death can occur. Thus, zoonotic transmission of Macacine alphaherpesvirus 1 (McHV1; formerly known as monkey B virus) from rhesus macaques to humans causes encephalitis with about 70% case-fatality rate [3].

Papiine alphaherpesvirus 2 (PaHV2) is closely related to McHV1 but has not been reported to cause disease in humans [3,4,5]. Herpes simplex viruses (HSV) 1 and 2 are more distant, with HSV2 showing a slightly closer overall relationship to PaHV2 than HSV1 [3,5]. Infection with PaHV2 appears to be common among olive and chacma baboons as documented by serological evidence [6]. In general, PaHV2 biology mirrors that of McHV1 in several respects, including neuropathogenesis in mice and sensitivity to antivirals [7,8], making it an interesting model virus for the study of zoonotic encephalitis caused by simplex viruses. Interestingly, two groups of PaHV2 strains exist, differing greatly with regard to neuropathogenicity in mice [9]. Interstrain hybrids have been used to map the neuropathogenicity determinant to the UL39 gene, which encodes the large subunit of the viral ribonucleotide reductase [10]. However, further molecular analysis of PaHV2 infection has been difficult due to the lack of a recombinant system.

Recombinant systems for viruses provide an opportunity to efficiently introduce alterations into the viral genome before rescuing the virus. Several methods have been applied to generate recombinant herpesviruses, including simplex viruses. First, virus was rescued from a set of overlapping cosmids, as first demonstrated for HSV1 [11]. More recently, fosmids, cosmids with a low-copy F-factor origin, have been used to recue viruses, including Cercopithecine alphaherpesvirus 2 (CeHV2) [12,13,14]. Second, bacterial artificial chromosomes have been widely applied to clone and modify herpesviral genomes [15,16,17]. Finally, transformation-associated recombination (TAR) in yeast has been applied to clone and assemble genomes of herpesviruses [18,19] and other viruses [20].

In this work we have established a recombinant system for PaHV2 employing fosmid cloning and TAR. We demonstrate rescue of wild-type and reporter viruses, which show growth characteristics similar to wild-type. Furthermore, reporter viruses were successfully applied to the study of cell susceptibility, antivirals and neutralization.

## 2. Materials and Methods

### 2.1. Plasmids, Oligonucleotides and Microorganisms

Plasmids pCC1FOS (Lucigen, Middleton, WI, USA) and YCplac22 [21] were used for fosmid cloning and TAR. For direct cloning of fragments, the multiple cloning site of pCC1FOS was modified by ligating annealed oligonucleotides mcs-FOS-f/mcs-FOS-r into pCC1FOS cut with EcoRI and SphI, to give pCC1FOS-B. Plasmids pcDNA3-EGFP-en [12], pcDNA3-Gluc-en and pHW2000GG-seg8-A/PR/8/34-M2A-Gluc-en [12] were used for recombineering. Plasmid pcDNA3-Gluc-en was constructed by amplification of the Gluc-cassette from pHW2000GG-seg8-A/PR/8/34-M2A-Gluc-en using primers Gluc-5A/Gluc-3StopN and insertion of the purified, Acc65I/NotI digested fragment into pcDNA3 (Invitrogen, Waltham, MA, USA). Oligonucleotides were ordered from Sigma-Aldrich (St. Louis, MO, USA) (see Table 1).

For cloning and recombineering, we used the *Escherichia coli* host strains DH10B [22], EPI300 (Lucigen, Middleton, WI, USA), GS1783 [23] and PMC103 [24]. The yeast strain *Saccharomyces cerevisiae* VL6-48N was used for TAR [25].

### 2.2. Cells and Virus

Vero76, Cos7 (both African green monkey (AGM), kidney), A549 (human, lung epithelial), 293T (human, kidney), HeLa (human, cervix epithelial), MamuK8639 (rhesus macaque, *Macaca mulatta*, kidney) [12], TeloRF (rhesus macaque, *Macaca mulatta*, fibroblast) [26] and LR7 (mouse fibroblast) [27] cells were cultivated in Dulbecco′s Modified Eagle′s Medium (DMEM) containing 10% fetal calf serum (FCS) and 100 U/mL penicillin and 100 μg/mL of streptomycin. The identity of human cell lines was verified by STR analysis using a published protocol [28]. For non-human cell lines, species identity was confirmed by partial sequencing of mitochondrial genes [29].

Papiine alphaherpesvirus 2 strain X313 was a kind gift by David Brown and Matthew Jones, Public Health England. Sequencing of this parental virus demonstrated that all coding regions were identical with the published sequence (DQ149153) [5] with a few exceptions bearing amino acid changes: UL21 P420A, UL29 T533A and US4 H427D. One additional nucleotide change within the coding region of UL44 did not affect the amino acid sequence. Some nucleotide changes were located outside the coding regions, and two extremely GC-rich regions within noncoding regions could not be resolved. The sequence of our strain has been deposited in Genbank (OM021999).

### 2.3. Fosmid Cloning

Fosmid cloning of PaHV2 DNA was performed as described previously [9]. Briefly, PaHV2 virions were collected from cell culture supernatant by ultracentrifugation (WX Ultra 80, Thermo Scientific Sorvall, Dreieich, Germany; Surespin rotor, 28,000 rpm, 70 min) and the virion pellet resuspended in 100 µL of PCR lysis buffer (50 mM of KCl, 10 mM of Tris pH 8.3, 1.5 mM of MgCl_2_, 0.001% gelatine, 0.5% triton X-100, 35 µg/mL of Proteinase K) followed by incubation for 1 h at 56 °C. The viral DNA was sheared 7 times through a 27G needle and precipitated after addition of 40 µL of 5 M NaCl and 125 µL of isopropanol. The DNA pellet was dissolved in 10 µL of H_2_O and processed (end repair) for fosmid cloning as recommended by the manufacturer (CopyControl Fosmid Library Production Kit; Lucigen, Middleton, WI, USA). After separation on a 0.8% low melting agarose gel, a gel slice encompassing DNA fragments of about 20–40 kbp was cut and DNA recovered by digesting the agarose with GELase. Ligation into 500 ng of Eco72I-linearized pCC1FOS vector was set up using 250 ng size-fractionated virion DNA and subsequently packaged into lambda particles using MaxPlax Packaging Extracts, according to the protocols supplied by the manufacturer. Finally, *E. coli* EPI300 cells were transduced with lambda particles and resulting colonies were screened by colony PCR to identify the region of the genome present in individual clones. Clones chosen for further analysis were partially sequenced (ends of the insert) and analyzed by restriction digest.

For direct cloning of pCC1FOS-B-HVP2-USUL (H-60), unsheared virus DNA was digested with NheI and SspI, separated on a low melting agarose gel, as described above. The DNA recovered from the gel was ligated into pCC1FOS-B cut with Eco72I and NheI, packaged into lambda particles and processed as described above.

### 2.4. Transformation-Associated Recombination

Cloning of missing fragments by TAR was essentially performed as described in [30,31]. As the vector backbone, we used YCplac22 [21], which was amplified with primers providing the targeting sequences (hooks) for recombination (see Table 1). Primer pairs TAR-HVP2-Mid-F/R, TAR-HVP2-Cent-F/R and TAR-HVP2-G-F/R were employed to produce target vectors for cloning TAR-HVP2-Mid, TAR-HVP2-Cent and TAR-HVP2-G, respectively.

For transformation, *S. cerevisiae* strain VL6-48N [25,31] was grown overnight (100 mL) to an OD_600_ of 2.0–2.5. Yeast cells were then washed in 1 M of sorbitol, followed by resuspension in 20 mL of SPE solution (1 M of sorbitol, 0.01 M of sodium phosphate, 0.01 M of Na_2_EDTA, pH 7.5). For spheroplast generation, 40 μL of 2-mercaptoethanol and 60 µL of Zymolyase stock solution (10 mg/mL zymolyase 20T in 25% (*w*/*v*) glycerol) were added, and cells were incubated at 30 °C with slow shaking (60 rpm). The optimal level of spheroplasting was determined by measuring OD600 of a 1:10 dilution in 1 M of sorbitol or 2% SDS (optimal ratio 3–5) [31]. Spheroplasts were carefully washed twice in 1 M of sorbitol and finally resuspended in 2.0 mL of STC solution (1 M of sorbitol, 0.01 M of Tris-HCl, 0.01 M of CaCl_2_, pH 7.5.). For transformation, 100 µL of spheroplast suspension were mixed with 0.5 µg of TAR cloning vector and 2 µg of sheared virus DNA. After 10 min incubation at room temperature, spheroplasts were gently mixed with 800 µL of PEG8000 solution (20% (*w*/*v*) PEG8000, 10 mM of CaCl_2_, 10 mM of Tris-HCl, pH 7.5) and incubated for 15 min at room temperature. Then, spheroplasts were collected by centrifugation and carefully resuspended in 800 µL of SOS solution (1 M of sorbitol, 6.5 mM of CaCl_2_, 0.25% yeast extract and 0.5% peptone), followed by incubation at 30 °C for 40 min. Finally, cells were mixed with sorbitol-containing Trp-dropout top agar and plated on sorbitol-Trp-dropout agar plates. Plates were incubated at 30 °C until colonies formed (about 5 days). Screening was performed by PCR on pools of colonies, followed by identification of individual positive clones from positive pools [31].

To produce DNA for transfection while preserving unstable regions, the plasmids were transferred from yeast to *E. coli* strain PMC103 [24], which has been described to provide increased stability of palindromic sequences. Positive clones were cultured at room temperature to further increase genetic stability [32].

### 2.5. Recombineering

For recombineering by en passant mutagenesis [33], required fosmids were transferred into *E. coli* host strain GS1783 [23], which allows for heat-inducible expression of phage recombinases and arabinose inducible expression of I-SceI. PCR fragments for recombineering were prepared using plasmids pcDNA3-EGFP-en [12], pcDNA3-Gluc-en and pHW2000GG-seg8-A/PR/8/34-M2A-Gluc-en [12] as templates as indicated in Table 2. PCR products were subsequently digested with DpnI to remove template. The final products harbored the amplified en passant cassette flanked by 50 bp sequences homologous to their respective target.

*E. coli* GS1783 strains harboring the fosmids HVP2a41 or HVP2a3-45 were prepared for electroporation as described before [12]. Briefly, cultures were grown at 30 °C to an optical density (600 nm) of 0.5–0.7 and transferred to a shaking water bath to induce recombinases during a 15 min incubation at 42 °C. Afterwards, cells were cooled in an ice water bath and washed three times in ice-cold sterile water, before the pellet was resuspended in an equal volume of 10% glycerol. PCR fragments were mixed with 100 µL of bacterial suspension and electroporated at 2500 V, 25 µF and 200 W in a Gene Pulser (Bio-Rad, Hercules, CA, USA), followed by addition of 1 mL of LB medium and incubation of 2 h at 30 °C. Recombinant fosmids were selected after plating cells on LB agar plates containing chloramphenicol and kanamycin. Colonies were analyzed by PCR for successful integration of the en passant cassette using primers positioned outside the region used for recombination. For subsequent removal of the kanamycin cassette from positive clones, 100 µL of an overnight culture were inoculated into 2 mL of LB medium containing 25 µg/mL of chloramphenicol and 1% L-arabinose and incubated at 30 °C for 1 h to induce *I-SceI* expression. Then, the culture was transferred to a shaking water bath at 42 °C for 30 min to induce phage recombinases. After an additional incubation at 30 °C for 1–2 h, dilutions (10^−3^ to 10^−4^) were plated on LB agar plates containing chloramphenicol and L-arabinose. Colonies negative for kanamycin were identified by colony PCR. The altered region was sequenced to confirm the desired changes.

### 2.6. Virus Rescue

Rescue of PaHV2 was performed as previously described for CeHV2 [12]. Briefly, Vero76 cells were seeded in 12-well plates at 10^5^ cells/well and transfected on the next day with a set of linearized fosmids/plasmids. Transfection was performed with Lipofectamine 2000 (Thermo Fisher, Waltham, MA, USA) according to the protocols of the manufacturer, using 1 µg per plasmid. Cultures were screened for cytopathic effects (rounded cells, syncytia), which usually appeared after 3 days. Supernatant was harvested when all cells showed signs of infection and used to generate virus stocks.

### 2.7. High-Throughput Sequencing and De Novo Assembly

High-throughput sequencing was performed to validate the constructs and rescued viruses. DNA was isolated from virions collected by ultracentrifugation. Particles were resuspended in lysis buffer (50 mM of KCl, 10 mM of Tris pH 8.3, 1.5 mM of MgCl_2_, 0.5% Tween 20, 40 µg/mL of proteinase K) and incubated at 56 °C for 1 h. After inactivation of proteinase K at 95 °C for 10 min, DNA was concentrated by ethanol precipitation. Sequencing libraries were prepared using the NEBNext Ultra II FS DNA Library Prep Kit for Illumina (New England Biolabs, Frankfurt, Germany) according to the manufacturer’s protocol. In addition, Betaine was used at a final concentration of 1 M during the indexing PCR to mitigate the sample’s high GC-content. The libraries were sequenced on an Illumina MiSeq using a 600v3 reagent kit generating 2 × 300 nt paired-end reads. Sequencing reads were trimmed using fastp [34] and de novo assembled using SPAdes [35]. Assemblies were compared to and annotated according to reference construct (DQ149153) using Geneious Prime 2021 (Biomatters, Auckland, New Zealand). The sequences of our parental virus strain and two rescued viruses (set M and set C) have been deposited in Genbank (OM021999, OM021998, OM021997).

### 2.8. Plaque Assay and Virus Growth Curves

To determine virus titers by plaque assay [12], Vero76 cells were seeded in 24-well plates at 100,000 cells/well. After overnight incubation cells, were infected with 10-fold serial dilutions of harvested virus-containing supernatant. Following 1 h of incubation at 37 °C, inoculum was removed and replaced by 0.5 mL of overlay medium containing 1% Avicel (FMC, Philadelphia, PA, USA). Plates were incubated for 3–4 days until plaques developed. For harvest, overlay medium was aspirated, and cells were washed once with phosphate-buffered saline (PBS) to remove remnants of Avicel. Following fixation with cold methanol for 10 min at −20 °C, plates were dried. To visualize plaques, cells were stained at room temperature with crystal violet solution (0.2% crystal violet, 20% ethanol) for 2 min and washed once with water. Plaques were counted, and virus titer was calculated as plaque-forming units per milliliter (pfu/mL).

Single-step growth curves were performed in 24-well plates as previously described [12]. Briefly, target cells (Vero76) were seeded at 50,000 cells/well, incubated overnight and then infected in triplicates with a multiplicity of infection (MOI) of 1 for 1 h. After infection, inoculum was removed, and cells were washed once with PBS and incubated in 0.5 mL of fresh medium. At fixed time points, supernatants were harvested, centrifuged to remove floating cells and debris and frozen at −80 °C for subsequent titration.

### 2.9. Microscopy

For microscopy, cells were seeded in plastic multi-well plates as detailed in sections on virus rescue or inhibitor assay. For time series, cells were seeded in lumox 96-well plates (Greiner, Kremsmünster, Austria) and incubated in the presence of cell-permeable nuclear counterstain Hoechst 33342 (Invitrogen, Karlsruhe, Germany). Microscopic imaging was performed on a Zeiss LSM800 employing 405 nm and 488 nm laser lines, 10× magnification and GaAsP detectors. Brightfield images were taken using the ESID module. Microscopic images were recorded using Zeiss ZEN software. Images were further processed (cropping, adjustment of brightness, scale bar) using Image J/Fiji [36].

### 2.10. Infection and Luciferase Assay

For infection kinetics, Vero76 cells were seeded in 96-well plates at 10,000 cells/well. On the next day, cells were infected in triplicates with PaHV2 cmvGluc or PaHV2 Gluc-2A-UL35 with MOI 1, 0.1 or 0.01. After 1 h, inoculum was removed, and cells were washed four times with PBS, to remove Gaussia luciferase (Gluc) present in the inoculum. After the final wash, 100 µL of DMEM containing FCS, penicillin and streptomycin was added. Samples of 25 µL of supernatant were collected immediately afterward (0 hpi, hours post infection), and every hour for the next eight hours, followed by 24, 48 and 72 hpi. All samples were frozen at −20 °C.

For multicycle infection, cells were seeded in 6-well plates at 250,000 cells/well. The next day, cells were infected in triplicates with PaHV2-cmvGluc with MOI 0.0004 (100 pfu/well). After 1 h, inoculum was replaced with DMEM containing FCS, penicillin and streptomycin. At certain intervals, samples of 25 µL of supernatant were collected and frozen at −20 °C.

To determine luciferase activities, samples of 25 µL of supernatant were collected in white opaque plates (Nunc/ThermoFisher, Waltham, MA, USA) and measured in a Plate Chameleon V (Hidex, Turku, Finland) instrument by injection of 50 µL assay buffer (DPBS w/o calcium, magnesium, containing 2 µM coelenterazin) and 1 s counting time after a 100-ms delay.

### 2.11. Inhibitor Assay

For inhibitor assay, we adapted a published protocol [37] to the use of luciferase-based detection of infection. Inhibitors were dissolved in DMSO (acyclovir, ACY) or in water (ganciclovir, GCV; cidofovir, CDV; foscarnet, FOS) in stock solutions of 4 mg/mL. We seeded Vero76 cells in 96-well plates at 5 × 10^4^ cells/well. On the next day, we prepared a dilution of PaHV2 Gluc-2A-UL35 virus to 500 pfu/well (MOI 0.01), as well as a two-fold dilution series of antiviral compounds, both in DMEM without FCS. In parallel, dilution series with diluents (DMSO or water) were performed. Cells were first preincubated for 1 h at 37 °C with the respective dilutions of the antiviral compounds. For infection, medium was aspirated and replaced by diluted virus. After incubation for 1 h at 37 °C, cells were washed at least once with PBS to remove Gluc present in the inoculum, followed by addition of diluted compound in a final volume of 200 µL DMEM without FCS. In addition, controls without virus (negative control) or without virus and compound (positive control) were prepared. All samples were set up in triplicates. After 48 h, 25 µL of cell culture supernatant was removed and assayed for Gluc activity. Sample values were corrected for background by subtracting the mean of negative control and normalized to the positive control.

### 2.12. Neutralization Assay

For neutralization assay, we adapted existing microneutralization protocols developed for herpesviruses and influenza viruses [38,39,40,41]. Blood serum samples were taken from olive baboons (*Papio anubis*) during the annual health monitoring. Sera was tested in a colony surveillance assay (CSA: Panel E Kit) (Intuitive Biosciences, Madison, WI, USA) detecting antibodies against primate simplex viruses among others [42]. We seeded Vero76 cells in 96-well plates at 5 × 10^4^ cells/well. On the next day, we prepared a dilution of PaHV2 Gluc-2A-UL35 virus to 2000 pfu/mL, as well as a 2-fold dilution series of heat-inactivated (30 min at 56 °C) sera, starting at 1:10. Diluted virus (100 pfu in 50 µL) and 50 µL of serum dilution were mixed, giving a 1:20 starting dilution, and incubated for 1 h at 37 °C. For infection, medium was aspirated, and cells were washed with PBS and subsequently incubated with the virus-serum mixtures. After 24 h, 25 µL of cell culture supernatant was removed and assayed for Gluc activity. Sample values were corrected for background by subtracting the mean of negative control and normalized to the positive control. Values were converted from percent infection to percent neutralization.

### 2.13. Statistical Analysis

Most data were analyzed and graphed using Excel and its functions to calculate mean and standard deviation (SD) or standard error of the mean (SEM). Statistical significance was calculated by the *t*-test. Data from neutralization or inhibitor assays were first corrected for background (luciferase activity of uninfected untreated cells) and normalized for infection (luciferase activity of infected untreated cells) using Excel. Statistical analysis of normalized data was done using Graphpad Prism using nonlinear regression (curve fit with least squares regression) employing a dose-response calculation for inhibitors assuming a variable slope (Hill equation < 0.0), as detailed in [43].

## 3. Results

### 3.1. Cloning of the Papiine Alphaherpesvirus 2 Genome

**Figure 1:**
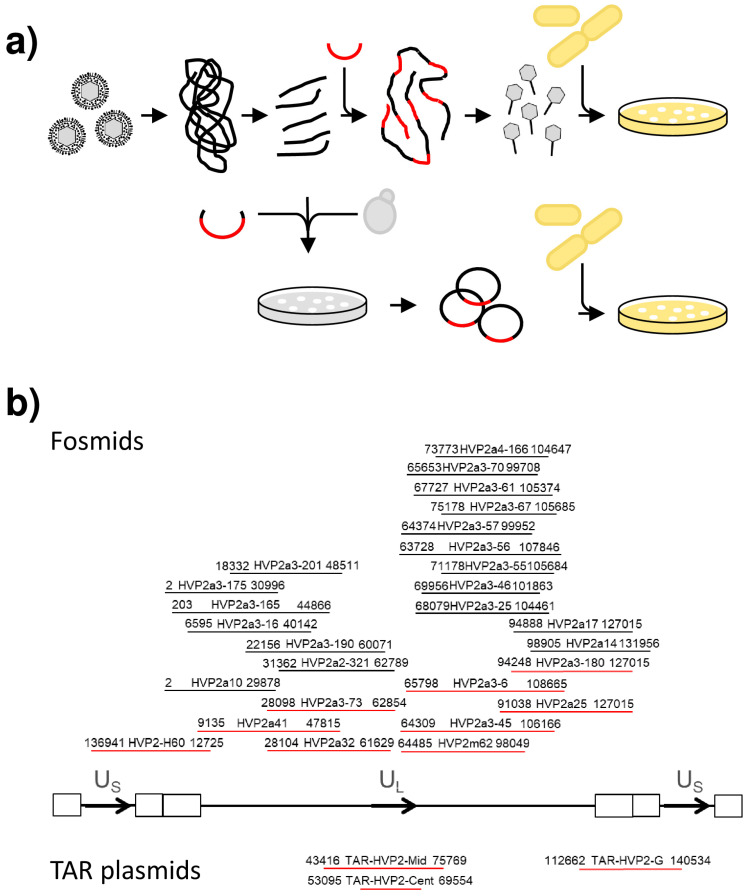
Cloning and characterization of PaHV2 plasmids. (**a**) Scheme of PaHV2 genome cloning (modified from [12]). Viral DNA was isolated from virus particles and sheared. For fosmid cloning (upper panels), viral DNA fragments were end-repaired and size fractionated on an agarose gel. Fractionated fragments were ligated into fosmid vector pCC1FOS (red) and packaged into phage lambda particles to transduce cells of *E. coli* strain EPI300. Individual clones were further characterized by colony PCR, end sequencing and restriction digest. For transformation-associated recombination (TAR) (lower panels), viral DNA fragments were mixed with a yeast shuttle vector harboring targeting sequences (hooks) for recombination and transformed into yeast strain VL6-48N. Positive clones were identified by colony PCR and subsequently transferred to *E. coli* for final characterization by restriction digest and end sequencing. (**b**) Overview of all genomic clones. A concatemeric section of the genome of PaHV2 is schematically represented, with US regions drawn on both sides of the UL region. Direction of gene order in UL and US regions is indicated by arrows and inverted repeats are drawn as boxes. Fosmid clones are shown above the genome representation, whereas TAR clones are shown below. Positions of genomic clones are drawn as lines. Names of the fosmids/plasmids and nucleotide positions of the insertions are given above the respective lines.

For cloning of the PaHV2 genome, we initially applied the same strategy as for CeHV2 [12]. DNA isolated from virus particles was fragmented, ligated into fosmid vector pCC1FOS and, after packaging into lambda particles, transduced into *E. coli* (Figure 1a). Individual clones were analyzed by PCR to map the approximate position on the viral genome, followed by sequencing of the insert ends to obtain the exact position of the inserts. Characterization by restriction digest was used as an initial test for integrity of the cloned inserts. In this way, we were able to obtain a number of clones spanning most of the UL and IRL regions (Figure 1b upper panel). However, clones containing US and IRS regions, as well as about 2 kb in the center of the UL region could not be recovered despite screening of more than 2000 clones.

We then attempted targeted restriction enzyme-based cloning of the missing fragments but could only recover one clone (HVP2-H60), covering the US-IRS-IRL-UL junction. Finally, we turned to TAR, to recover the still missing regions as yeast artificial chromosomes in a YCplac22 vector. After transfer of clones from yeast into *E. coli*, plasmids were further characterized, including full sequencing. The initial sequencing results pointed out deletions in three regions of the genome, the center of UL as well as the two IRS regions, affecting the palindromic origin sequences. Therefore, we turned to use *E. coli* strain PMC103 for transfer of DNA from yeast, since this strain shows increased stability of palindromic sequences [24].

**Figure 2:**
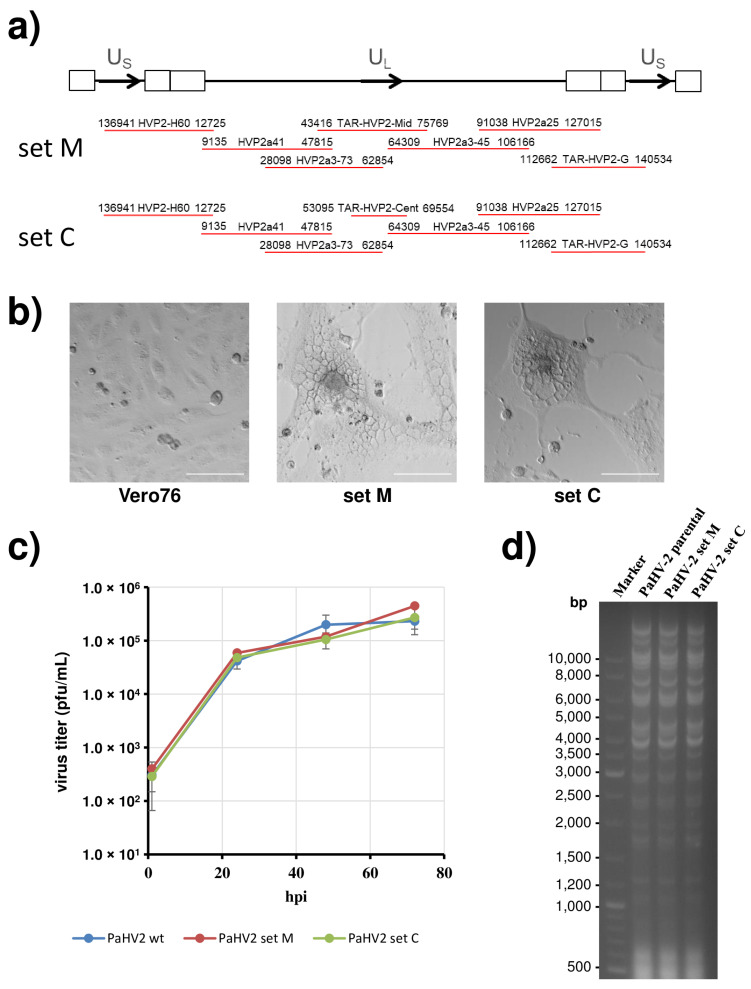
Rescue of PaHV2 from plasmids. (**a**) Schematic depiction of the PaHV2 genome along with two sets of fosmids/TAR plasmids used for rescue. Identical fosmids were used for both sets except for TAR-HVP2-Mid (set M) or TAR-HVP2-Cent (set C). (**b**) Brightfield images of untransfected Vero76 cells (left panel), or Vero76 cells transfected with set M (middle panel) or set C (right panel) taken at 3 days after transfection. Images were taken at 10× magnification. The scale bar represents 100 µm. (**c**) Replication kinetics of parental PaHV2 and recombinant PaHV2, derived from two sets of plasmids, on Vero76 cells infected with MOI 1. The average of three independent experiments, each performed with triplicate samples is shown. Error bars represent standard error of the mean (SEM). (**d**) Restriction digest of viral genomes. Viral DNA isolated from particles of parental or recombinant PaHV2 (set M and set C) was digested with BamHI and subjected to gel electrophoresis. Sizes of marker bands are given on the left.

In the next step, two sets of five fosmid and two TAR clones each (set M and set C) were chosen for the rescue of infectious PaHV2 (Figure 2a). The plasmids were first linearized using unique restriction enzyme sites in the multiple cloning sites flanking the insert or in the vector backbone and then transfected into Vero76 cells. Cytopathic effects (syncytia formation) could be observed as early as 2 days after transfection (Figure 2b middle and right panels), while no changes were detected in untransfected Vero76 cells (Figure 2b left panel). Infectious virus could be transferred with the supernatant to uninfected cells, and PCR confirmed its identity as PaHV2.

To further characterize the rescued viruses, we compared parental and recombinant viruses in a growth analysis on Vero76 cells infected at MOI 1. As shown in Figure 2c, both recombinant viruses (derived from set C or set M) produced progeny viruses to the same extent and with the same kinetics as the parental virus. Finally, we prepared viral DNA from virus particles and performed restriction digest (Figure 2d). All three genomes showed an identical pattern indicating no major alterations in genome structure. In parallel, DNA was subjected to next generation sequencing. Apart from a few highly GC-rich stretches, which could not be resolved, sequences were almost identical to the parental PaHV2 X313 genome, clearly demonstrating no deletions or frameshift mutations within coding regions. We note a single amino acid change with respect to our parental virus, which was due to UL13 A424T and was also present in fosmid HVP2a41. Importantly, we did not detect any deletions affecting palindromic origin sequences. Thus, we were able to demonstrate successful rescue of PaHV2 from a plasmid-based recombinant system.

### 3.2. PaHV2 Genomes with Reporter Genes

**Figure 3:**
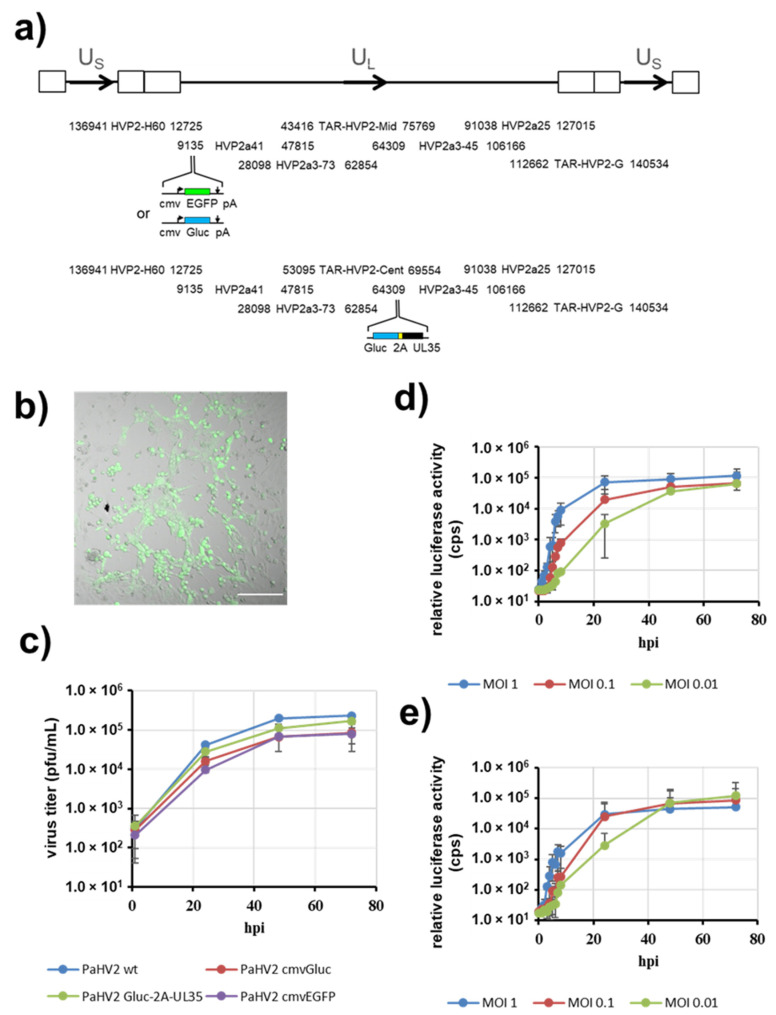
Rescue of PaHV2 bearing reporter genes. (**a**) Schematic depiction of the PaHV2 genome along with the sets of fosmids/TAR plasmids used for rescue. Reporter cassettes introduced by recombineering are indicated. (**b**) Microscopic representation of a plaque formed by PaHV2-cmvEGFP three days after transfection. Brightfield and fluorescent images are merged. The scale bar represents 200 µm. (**c**) Replication kinetics of parental PaHV2 and recombinant PaHV2 bearing reporter cassettes on Vero76 cells infected with MOI 1. The average of two (PaHV2 Gluc-2A-UL35) or three (all other viruses) independent experiments, each performed with triplicate samples is shown. Error bars represent SEM. (**d**,**e**) Kinetics of luciferase activity of PaHV2-cmvGluc (**d**) or PaHV2-Gluc-2A-UL35 (**e**) in Vero76 cells infected at the indicated MOIs. At the indicated time points after infection cell culture supernatant was harvested and luciferase activity determined. The averages of two to four independent experiments, each performed with triplicate samples, are shown. Error bars represent SEM.

In a next step, we wanted to insert reporter genes into the viral genome. To obtain viruses with immediate expression of the reporter genes (Figure 3a), we chose to insert a reporter gene cassette into the intergenic region between UL3 and UL4, which has previously been used in the context of herpes simplex virus 1 for gene insertion [16,44]. Our reporter cassettes were driven by the human cytomegalovirus enhancer/promoter and contained either Gaussia luciferase (Gluc) or enhanced green fluorescent protein (EGFP) as reporter, followed by a bovine growth hormone polyadenylation signal. The cassettes were introduced into fosmid HVP2a41 by en passant mutagenesis [23] and controlled by PCR and sequencing of the modified regions. In addition, we fused the gene for Gluc with the 5′-end of the late gene UL35 in the context of fosmid HVP2a3-45. In this construct, both genes are linked with a porcine teschovirus 1 2A “Stop-Go” sequence enabling the processing into separate proteins [45,46].

For rescue of reporter viruses, the wild-type fosmids were replaced by the respective modified fosmids (Figure 3a). After transfection into Vero76 cells, clear cytopathic effects developed within 3–5 days, demonstrating rescue of PaHV2-cmvGluc, PaHV2-cmvEGFP and PaHV2-Gluc-2A-UL35 reporter viruses. Microscopic inspection of plaques formed by PaHV2-cmvEGFP clearly showed expression of EGFP (Figure 3b). The rescued viruses were passaged and subjected to a growth analysis. For this, Vero76 cells were infected with parental PaHV2 and the recombinant reporter viruses at MOI 1. All reporter viruses showed replication similar to parental PaHV2 (Figure 3c). The titers of the reporter viruses were slightly lower than parental PaHV2, but this deviation was not significant except for PaHV2 cmvEGFP at 72 hpi (Student’s *t*-test *p* = 0.040).

For further analysis, we focused on the reporter viruses carrying Gluc as a reporter, since this luciferase molecule is secreted into the cell culture supernatant and allows for highly sensitive detection and continuous sampling. We first determined the kinetics of Gluc expression on Vero76 cells infected with different MOI (1, 0.1 or 0.01). For PaHV2-cmvGluc infection at MOI 1 (Figure 3d), a first increase of Gluc activity could be detected as early as 2 hpi, and Gluc activity reached levels close to plateau around 6–8 hpi. When infected with lower MOI, the kinetics of Gluc expression was delayed and dose-dependent. However, in all conditions, maximum Gluc activity could be detected no later than 48 hpi. For PaHV2-Gluc-2A-UL35 (Figure 3e), where Gluc expression is coupled to late gene UL35, the kinetics of Gluc expression was delayed compared to PaHV2-cmvGluc, showing a first rise in Gluc activity between 4–6 hpi when infected at MOI 1. For this virus, Gluc activity was also dose dependent and maximum Gluc activity were detected no later than 48 hpi. Thus, we were able to generate reporter viruses allowing the sensitive detection of PaHV2 infection.

**Figure 4:**
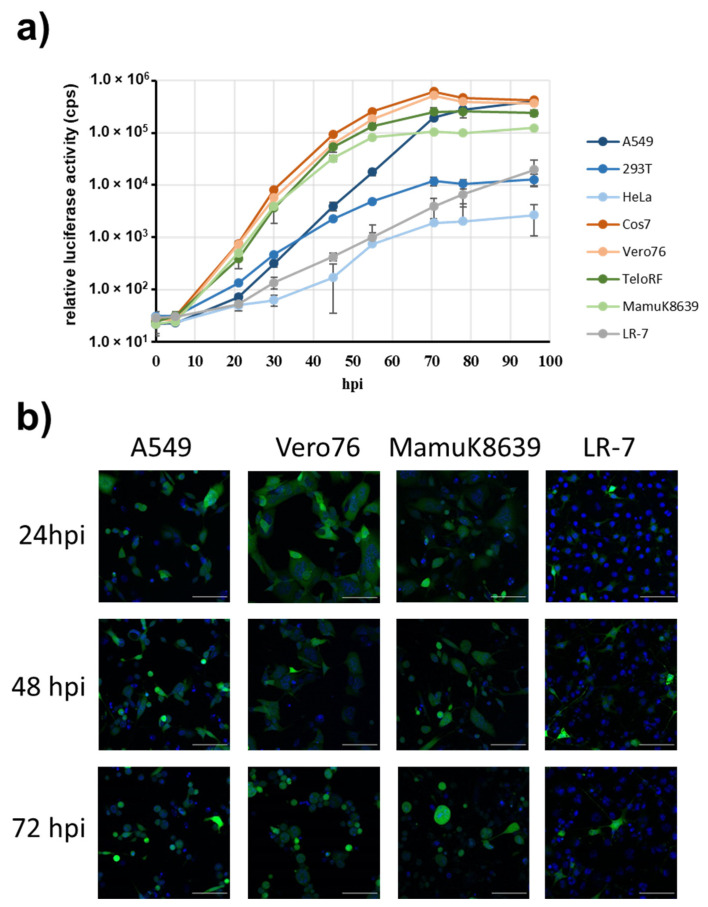
Susceptibility of cell lines. (**a**) Multicycle replication kinetics in cells of human (A549, 293T, HeLa), AGM (Vero76, Cos7), rhesus macaque (TeloRF, MamuK8639) or murine (LR-7) origin. Cells seeded in 6-well plates were infected with PaHV2-cmvGluc at low virus dose (100 pfu; MOI 0.0004) and small aliquots of cell culture supernatant collected over the course of 96 h followed by measurement of luciferase activity. The results of a single, representative experiment are shown, which was confirmed in two separate experiments. Error bars represent SD of triplicate samples. (**b**) Microscopic images of representative human (A549), AGM (Vero76), rhesus macaque (MamuK8639) and murine (LR-7) cell lines infected with PaHV2-cmvEGFP at MOI 1. Cells were incubated with cell permeable nuclear stain Hoechst 33342, and confocal microscopic images were taken at 24, 48 and 72 hpi at 10× magnification. Shown are merged images in which fluorescence from EGFP is colored green, while Hoechst-derived fluorescence is colored blue. The scale bars represent 100 µm.

We next employed the reporter viruses for analysis of cell susceptibility of PaHV2. For this, we infected representative human (A549, 293T HeLa), AGM (Vero76, Cos-7), rhesus macaque (TeloRF, MamuK8639) or murine (LR-7) cell lines with PaHV2-cmvGluc at a low dose (MOI 0.0004). Cell culture supernatants were continuously sampled up to 96 hpi, to allow for multicycle replication, and luciferase activity was determined. As shown in Figure 4a, PaHV2-cmvGluc growth is highly robust on Vero76 or Cos7 cells, derived from AGM, and also TeloRF and MamuK8639 cells from rhesus macaques. Growth on human cell lines was more variable, with A549 cells performing best, while replication in HeLa cells was clearly compromised. The murine cell line LR-7 also supported growth of PaHV2-cmvGluc, but at a low level. In parallel, we infected selected cell lines with PaHV2-cmvEGFP at MOI 1 to detect cell susceptibility at the single cell level. At 24 hpi, EGFP expression was clearly detected and slightly increased at 48 and 72 hpi (Figure 4b). Cell lines supporting efficient replication, such as A549, Vero76 and MamuK8639, demonstrated infection of most of the cells and also signs of CPE, such as syncytia formation and chromatin marginalization [47]. EGFP fluorescence was located throughout the cell, but more intense in the cytoplasm. In addition, fluorescence appeared to be less intense in large syncytia, as observed for infected Vero76 and MamuK8639 cell. In contrast, for the murine LR-7 cells, we observed few infected cells, no syncytia formation or chromatin marginalization and apart from rare local foci infection did not spread to other cells.

### 3.3. Inhibition of Reporter Virus Infection by Antibodies and Antivirals

**Figure 5:**
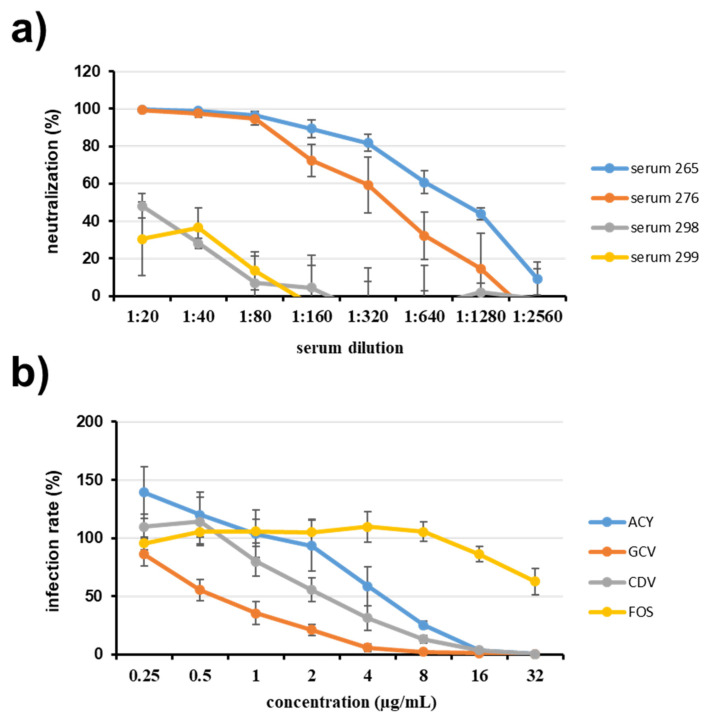
Inhibition of virus infection. (**a**) Neutralization assay. Virus (PaHV2-cmvGluc) and serum dilutions were preincubated for 1 h and then added to Vero76 cells in 96-well plates. After 24 h, activity of Gluc was assessed in the supernatant. Sera were from olive baboons (Papio anubis) previously tested positive (sera 265, 276) or negative (sera 298, 299) for antibodies against PaHV2 in a colony surveillance assay. The average of three independent experiments performed with triplicate samples is shown. Error bars represent SEM. (**b**) Antiviral inhibition assay. Vero76 cells seeded in 96-well plates were preincubated for 1 h with serial dilutions of antivirals acyclovir (ACY), ganciclovir (GCV), cidofovir (CDV) or foscarnet (FOS), followed by infection with PaHV2-Gluc-2A-UL35. After infection culture in presence of antiviral continued until 48 hpi, Gluc activity was measured from the supernatant. The average of three independent experiments, each performed with triplicate samples, is shown. Error bars represent SEM.

In a final set of experiments, we wanted to demonstrate the use of our reporter viruses for detecting inhibition of virus replication. First, we measured antibody-mediated neutralization of infection using sera from *Papio anubis*. For this, we set up a microneutralization assay, in which 100 pfu of PaHV2-cmvGluc were mixed with dilutions of sera. We chose two sera from animals tested positive for antibodies against PaHV2 in a commercially available colony surveillance assay and two sera from negative animals. Mixtures of virus and serum were used to infect Vero76 cells and luciferase levels determined after 24 h. As shown in Figure 5a, the sera from seropositive animals (265, 276) clearly inhibited infection, while sera from seronegative animals (298, 299) were not effective. When luciferase activities were measured after 48 h, the assay changed into a binary readout (infection yes or no), where complete inhibition was detected for 1:20 (serum 276) or 1:40 (serum 265) dilutions, while for sera from seronegative animals, virus replication could be detected for all tested dilutions.

Next, we measured the effect of known antivirals effective against herpesviruses. In these experiments we employed PaHV2-Gluc-2A-UL35, since Gluc expression in this virus is coupled to expression of the late gene UL35. Thus, inhibitory effects of the antivirals acyclovir (ACY), ganciclovir (GCV), cidofovir (CDV) or foscarnet (FOS), which target herpesviral replication, should be detected with this virus. We preincubated Vero76 cells with inhibitor dilutions, infected with PaHV2-Gluc-2A-UL35 (MOI 0.01) and then continued incubation in the presence of inhibitor dilutions until luciferase activity was measured at 48 hpi. As demonstrated in Figure 5b, clear inhibition of infection by ACY, GCV and CDV was observed, while FOS seemed to have only a slight inhibitory effect at the highest concentrations tested. The EC50 values determined for ACY (4.88 µg/mL, 95%CI: 3.14–7.76), GCV (0.67 µg/mL, 95%CI: 0.55–0.82) and CDV (2.48 µg/mL, 95%CI: 1.80–3.48) were slightly lower than determined by plaque assay [8]. In summary, we developed reporter viruses suitable for neutralization and inhibitor assays, which will be useful tools in screening experiments or comparative drug evaluation.

Overall, we were able to establish a recombinant system for PaHV2, which will be useful in comparative research on simplex viruses.

## 4. Discussion

Non-human primate (NHP) simplex viruses are considered to be similar to herpes simplex virus, but a few notable differences have been uncovered. For a detailed comparison on the molecular level, recombinant systems are required, but presently lacking for most NHP simplex viruses. We have previously succeeded in setting up such a system for CeHV2 [9], and now report a successful rescue of PaHV2.

In the development of the recombinant system for PaHV2, several obstacles had to be overcome. For one, it was not possible to clone parts of US-region using the fosmid methodology. This problem was solved by applying TAR to clone the missing regions. Sequencing of several clones used in initial rescue attempts uncovered deletions in palindromic regions of replication origins, which could be reduced by employing *E. coli* strains with increased palindromic stability [21]. The PaHV2 system, in its current form, still has some limitations. For instance, the TAR-based plasmids in their present form are high-copy plasmids in *E. coli*, making it difficult to apply recombineering. This problem will be solved in the future by conversion to plasmids bearing low copy F factor origins [42]. Recombineering will also help in reducing the extent of fragment overlapping, to make a larger region of the genome accessible to straightforward modification.

For sensitive detection and monitoring of infection, we developed several reporter viruses. For this, we inserted expression cassettes for Gluc and EGFP driven by the HCMV enhancer/promoter into the UL3/4 locus. PaHV2-cmvGluc allowed sensitive detection of infection, where values 100-fold over background were reached after 6 hpi, rendering it a useful tool to quantify infection. PaHV2-cmvEGFP allowed for localization of infected cells, but detection was much less sensitive than with the Gluc-expressing virus, allowing detection after 24 h. Part of this may be explained by the highly sensitive detection of Gluc in an enzymatic assay as compared to the non-enzymatic detection of EGFP [48]. In addition, EGFP has been reported to have limited stability in mammalian cells, while Gluc is highly stable [49,50]. Optimization of promoter or fluorescent protein may allow for a more sensitive detection and will make this an interesting system for single cell analysis, e.g., by flow cytometry. Using PaHV2-cmvGluc, we could demonstrate that the spectrum of cells lines susceptible to infection by PaHV2 appears to be broader than previously observed for CeHV2 [12]. In particular, PaHV2 demonstrated efficient growth in cells derived from rhesus macaques.

In addition, we were able to assess neutralization by sera from olive baboons. Sera from adult or aged animals (15 and 19 years) demonstrated inhibition of infection, while sera from infant animals (<1 year) were not effective, in agreement with in-house chip-based measurements of seropositivity and the published age-dependent seropositivity of baboons [51,52]. Complete neutralization was detected for 1:20 and 1:40 serum dilutions, a range also detected with microneutralization assays for HSV1 and NHP simplex viruses [40,53,54]. Because of the cross-reactivity of anti-PaHV2 antibodies with McHV1 [55,56], the assay could be used as an alternative and sensitive means to detect the presence of neutralizing antibodies against McHV1 in macaques, although this will require an in-depth analysis.

To analyze the effect of antiviral agents, we developed a reporter virus, PaHV2-Gluc-2A-UL35, in which Gluc was co-expressed with the late gene UL35 to allow measurement of inhibition of virus replication. We could demonstrate inhibition of PaHV2 replication by acyclovir, ganciclovir and cidofovir, but not foscarnet, in line with published data [8]. The IC50 values determined in our assay were somewhat lower compared to published results determined with focus reduction assay. This difference may be explained by the different mode of measurement (late gene expression vs. plaque formation) and the different mode of virus spread (free spread vs. restricted, local spread). Overall, our reporter virus constitutes a highly sensitive assay system, which allows for automatized measurement.

Collectively, we feel that the recombinant system described here will be valuable in the analysis of neuropathogenicity of simplex viruses. Several genes have been implicated in neuropathogenesis, with ICP34.5 believed to be the most important factor for neuroinvasion of HSV1 and HSV2 [1,57]. However, in contrast to simplex viruses from hominid primates [58], simplex viruses from cercopithecid primates (Old World monkeys) lack an ICP34.5 homolog [5,59,60], and at present, it is not clear whether its function was substituted by a different gene. Neuropathogenesis after zoonotic transmission may therefore depend on different virulence genes. Indeed, for neuropathogenesis of PaHV2 in mice, it has been reported that the viral ribonucleotide reductase large subunit (UL39) has a major role, based on analysis of neurovirulent and apathogenic strains [10]. However, a detailed molecular analysis to determine the specific function of UL39 driving neurovirulence has not been performed. The recombinant system for PaHV2 described here will enable such studies and thus make a valuable contribution to the comparative analysis of primate simplex virus neuropathogenesis.

In summary, we developed a recombinant system for PaHV2 and reporter viruses, which will be useful in the analysis of infection, neutralization and antiviral activity. Finally, the system will also prove invaluable in the analysis of determinants of PaHV2 neuropathogenicity.

## Figures and Tables

**Table 1:** Oligonucleotides used for cloning, TAR and recombineering.

Oligonucleotide Name	Oligonucleotide Sequence (Given in 5′-3′ Direction)
Oligonucleotides used for cloning	
mcs-FOS-f	AATTCCTCGAGGCTAGCTTAATTAAGGATCCCACGTGGGATCCATCGATACGCGTCGTACGGCATG
mcs-FOS-r	CCGTACGACGCGTATCGATGGATCCCACGTGGGATCCTTAATTAAGCTAGCCTCGAGG
Gluc-5A	CCGGTACCATGGGAGTCAAAGTTCTG
Gluc-3StopN	CCGCGGCCGCTTAGTCACCACCGGCCCCCTTG
Oligonucleotides used for TAR	
TAR-HVP2-Mid-F	GTAGTGCGCGTCCGCCACCAGCCCCAGCACCGTGTTGGTCGCCGTCTGGAGATCCTC-TAGAGTCGACCTGCAG
TAR-HVP2-Mid-R	GCGGTCTATGTGCTTCACCTGCACGAACTCGCTCACGGTGGTGCGCTTGGGTTTAAACGTCGTGACTGGGAAAACCCTG
TAR-HVP2-Cent-F	CAGCTCGCCGCAGAGCGACTCGTTAAGAGCCAGGAGGTCGGGGTCGAAGGATCCTC-TAGAGTCGACCTGCAG
TAR-HVP2-Cent-R	GCGACACCACCGCCGATCGACCCGCTGTGGAAACCACACGCACATAGACGTTTAAA-CGTCGTGACTGGGAAAACCCTG
TAR-HVP2-G-F	GGCTCGCGTTCGTGATCATCACCGTCATGGTGCTGCGGCCATGCCGTCCGATCCTCTAGAGTCGACCTGCAG
TAR-HVP2-G-R	CCGTGCTGGCGATCAGCCCCTGGCTCGCCACGCGCAGCAGGATCTCGCAGTTTAAACGTCGTGACTGGGAAAACCCTG
Oligonucleotides used for recombineering	
ep-HVPUL4pA-cmv	TCCCGCGGCCCCGGGCGCTCCAGAGACGCGGCGAGACGAATAAACGCGGTGTTGACATTGATTATTGACT
ep-HVPUL3-gGHpA	CGCCTCGCCGATCCCAAGGATGGATGACACACGAATAAATATTTCAAACGTCCCCAGCATGCCTGCTATT
ep-Pa35-Gluc-5	TCCGCCGCCCTCCTCCCGGTCGCCGTTGCGCCCCGCCCGCCGCCCGCGCCATGGGAGTCAAAGTTCTGTTT
ep-Pa35-2A-3	TCGGGGTGAAGCGTGTCGGGCCGGTGGAACTGCGGGACCGCGGCGGACATCGGGCCCGGGTTTTCTTCCAC

**Table 2:** Recombineering. Templates, primers and target fosmids used for recombineering.

Virus Construct	Fosmid	Template	Primer
HVP2-cmvGluc	HVP2a41	pcDNA3-Gluc-en	ep-HVPUL4pA-cmv ep-HVPUL3-gGHpA
HVP2-cmvEGFP	HVP2a41	pcDNA3-EGFP-en	ep-HVPUL4pA-cmv ep-HVPUL3-gGHpA
HVP2-Gluc-2A-UL35	HVP2a3-45	pHW2000GG-seg8-A/PR/8/34-M2A-Gluc-en	ep-Pa35-Gluc-5 ep-Pa35-2A-3

## Data Availability

The data presented in this study are available on request from the corresponding author.
